# *catena*-Poly[[[bis­(thio­urea-κ*S*)copper(I)]-μ-thio­urea-κ^2^ *S*:*S*] iodide acetonitrile hemisolvate]

**DOI:** 10.1107/S1600536808007265

**Published:** 2008-03-20

**Authors:** Li Jia, Ling-Qian Kong, Da-Cheng Li

**Affiliations:** a School of Chemistry and Chemical Engineering, Liaocheng University, Shandong 252059, People’s Republic of China; b Liaocheng Vocational and Technical College, Liaocheng, Shandong 252000, People’s Republic of China; c Dongchang College of Liaocheng University, Liaocheng, Shandong 252000, People’s Republic of China

## Abstract

The title complex, {[Cu(CH_4_N_2_S)_3_]I·0.5CH_3_CN}_*n*_, was formed by the reaction of CuI and thio­urea in acetonitrile. There are two independent Cu^I^ ions in the asymmetric unit which are coordinated by two terminal and two bridging thio­urea ligands to form a one-dimensional helical chain structure progagating in the *a*-axis direction. Each Cu^I^ ion is in a distorted tetra­hedral coordination environment. The crystal structure is stabilized by weak N—H⋯S and N—H⋯I hydrogen bonds.

## Related literature

For related literature, see: Bombicz *et al.* (2004 ▶); Bott *et al.* (1998 ▶); Stocker *et al.* (1996 ▶).
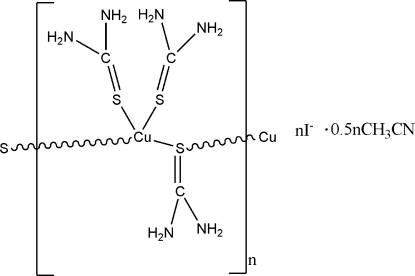


## Experimental

### 

#### Crystal data

[Cu(CH_4_N_2_S)_3_]I·0.5C_2_H_3_N
*M* *_r_* = 439.33
Orthorhombic, 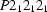

*a* = 13.392 (8) Å
*b* = 13.874 (9) Å
*c* = 15.289 (9) Å
*V* = 2841 (3) Å^3^
*Z* = 8
Mo *K*α radiation
μ = 4.14 mm^−1^
*T* = 298 (2) K
0.43 × 0.39 × 0.31 mm

#### Data collection

Bruker SMART CCD diffractometer
Absorption correction: multi-scan (*SADABS*; Sheldrick, 1996 ▶) *T*_min_ = 0.269, *T*_max_ = 0.360 (expected range = 0.207–0.277)
14883 measured reflections
4963 independent reflections
4175 reflections with *I* > 2σ(*I*)
*R*_int_ = 0.058

#### Refinement

*R*[*F* ^2^ > 2σ(*F* ^2^)] = 0.034
*wR*(*F* ^2^) = 0.077
*S* = 1.00
4963 reflections
280 parameters
H-atom parameters constrained
Δρ_max_ = 0.75 e Å^−3^
Δρ_min_ = −0.56 e Å^−3^
Absolute structure: Flack (1983 ▶), 2149 Friedel pairs
Flack parameter: −0.01 (2)

### 

Data collection: *SMART* (Bruker, 1997 ▶); cell refinement: *SAINT* (Siemens, 1996 ▶); data reduction: *SAINT*; program(s) used to solve structure: *SHELXS97* (Sheldrick, 2008 ▶); program(s) used to refine structure: *SHELXL97* (Sheldrick, 2008 ▶); molecular graphics: *SHELXTL* (Sheldrick, 2008 ▶) and *DIAMOND* (Brandenburg & Berndt, 2006 ▶); software used to prepare material for publication: *SHELXTL*.

## Supplementary Material

Crystal structure: contains datablocks I, global. DOI: 10.1107/S1600536808007265/lh2596sup1.cif

Structure factors: contains datablocks I. DOI: 10.1107/S1600536808007265/lh2596Isup2.hkl

Additional supplementary materials:  crystallographic information; 3D view; checkCIF report

## Figures and Tables

**Table 1:** Selected bond lengths (Å)

Cu1—S3	2.275 (2)
Cu1—S2	2.309 (2)
Cu1—S1	2.382 (2)
Cu1—S6^i^	2.411 (2)
Cu2—S4	2.299 (2)
Cu2—S5	2.335 (2)
Cu2—S1	2.341 (2)
Cu2—S6	2.435 (2)

Symmetry code: (i) 
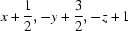
.

**Table 2:** Hydrogen-bond geometry (Å, °)

*D*—H⋯*A*	*D*—H	H⋯*A*	*D*⋯*A*	*D*—H⋯*A*
N1—H1*A*⋯I2^ii^	0.86	2.97	3.799 (7)	161
N1—H1*B*⋯S6^i^	0.86	2.68	3.524 (7)	167
N2—H2*A*⋯I2^ii^	0.86	3.14	3.929 (6)	154
N2—H2*B*⋯S5	0.86	2.96	3.752 (7)	154
N2—H2*B*⋯I1^iii^	0.86	3.17	3.667 (7)	119
N3—H3*A*⋯I2	0.86	2.87	3.645 (6)	150
N3—H3*B*⋯I1^iv^	0.86	3.06	3.720 (6)	135
N4—H4*A*⋯I2	0.86	3.11	3.837 (7)	144
N4—H4*A*⋯I1	0.86	3.22	3.706 (6)	119
N4—H4*B*⋯S6^i^	0.86	2.73	3.563 (7)	165
N5—H5*A*⋯N13^v^	0.86	2.41	3.192 (10)	152
N5—H5*A*⋯I2^iv^	0.86	3.32	3.796 (8)	118
N7—H7*A*⋯I1^vi^	0.86	3.04	3.839 (7)	156
N7—H7*B*⋯I2^iv^	0.86	3.02	3.837 (7)	159
N8—H8*A*⋯I1^vi^	0.86	2.93	3.759 (6)	161
N8—H8*B*⋯S5	0.86	2.69	3.526 (6)	166
N9—H9*A*⋯I2^vii^	0.86	3.12	3.922 (6)	155
N9—H9*B*⋯S4	0.86	2.61	3.429 (7)	161
N10—H10*A*⋯I1^vii^	0.86	3.01	3.716 (7)	141
N11—H11*A*⋯I1^viii^	0.86	2.99	3.791 (6)	155
N11—H11*B*⋯S2^iii^	0.86	2.63	3.466 (6)	163
N12—H12*A*⋯I1^viii^	0.86	2.92	3.732 (6)	158
N12—H12*B*⋯S1	0.86	2.54	3.338 (6)	155
N6—H6*A*⋯S2^v^	0.86	2.89	3.397 (6)	119
N6—H6*A*⋯N13^v^	0.86	2.51	3.266 (11)	147

Symmetry codes: (i) 
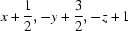
; (ii) 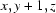; (iii) 
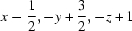
; (iv) 
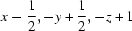
; (v) 
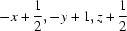
; (vi) 
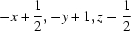
; (vii) 
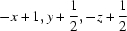
; (viii) 
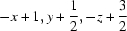
.

**Fig. 1.**
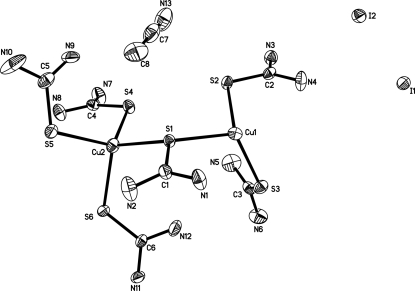
The asymmetric unit with atom labels and 30% probability displacement ellipsoids. H atoms are not shown.

**Fig. 2.**
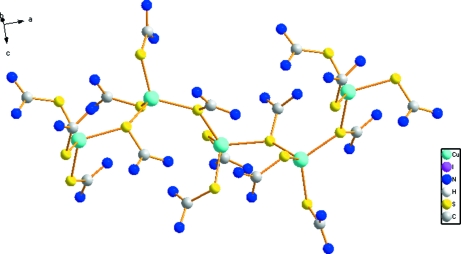
Part of the one-dimensional helical chain structure of the title complex.

## supplementary crystallographic information

### Comment

Some copper(I) compounds containing thiourea ligands have been described previously (Bombicz *et al.*, 2004; Bott *et al.*, 1998; Stocker *et al.*, 1996). In this paper, we report the synthesis and the structure of a complex formed by the reaction of thiourea with cuprous iodide. The asymmetric unit of the title compound is shown in Fig. 1. The Cu^I^ ions have distorted tetrahedral coordination geometries formed by two bridging thiourea ligands and two terminal thiourea ligands. A one-dimensional helical chain structure parallel to the *a* axis direction is formed (Fig. 2). An iodide counter ion and half an acetonitrile solvent molecule complete the formula unit although there are two formula units in the asymmetric unit of the crystal structure. The Cu—S distances are in the range of 2.275 (2)–2.435 (2) Å, and agree with those in related structures (Bombicz *et al.*, 2004). In the title compound, the S=C distances are the same within experimental error.

In the crystal structure, there are two different types of hydrogen bonds. Intramolecular N—H···S interactions appear to influence the conformation of the helical chains while intermolecular N—H···S and N—H···I interactions stabilize the crystal structure.

### Experimental

CuI (0.19 g 1 mmol) and thiourea (0.16 g 2 mmol) in 10 ml acetonitrile were refluxed for 24 h, forming a colorless solution. After filtration, the solution was allowed to evaporate slowly and crystals suitable for X-ray diffraction were obtained after several days.

### Refinement

All H atoms were placed geometrically and treated as riding on their parent atoms, with, N—H 0.86, C—H 0.96 Å, with *U*_iso_(H) = 1.2*U*_eq_(N), *U*_iso_(H) = 1.5*U*_eq_(C).

### Crystal data

**Table:**

[Cu(CH_4_N_2_S)_3_]I·0.5C_2_H_3_N	*F*_000_ = 1704
*M**_r_* = 439.33	*D*_x_ = 2.055 Mg m^−^^3^
Orthorhombic, *P*2_1_2_1_2_1_	Mo *K*α radiation λ = 0.71073 Å
Hall symbol: P 2ac 2ab	Cell parameters from 6067 reflections
*a* = 13.392 (8) Å	θ = 2.4–24.6º
*b* = 13.874 (9) Å	µ = 4.14 mm^−^^1^
*c* = 15.289 (9) Å	*T* = 298 (2) K
*V* = 2841 (3) Å^3^	Block, colorless
*Z* = 8	0.43 × 0.39 × 0.31 mm

### Data collection

**Table:**

Bruker SMART CCD diffractometer	4963 independent reflections
Radiation source: fine-focus sealed tube	4175 reflections with *I* > 2σ(*I*)
Monochromator: graphite	*R*_int_ = 0.058
*T* = 298(2) K	θ_max_ = 25.0º
φ and ω scans	θ_min_ = 2.0º
Absorption correction: multi-scan(SADABS; Sheldrick, 1996)	*h* = −15→15
*T*_min_ = 0.269, *T*_max_ = 0.360	*k* = −14→16
14883 measured reflections	*l* = −18→18

### Refinement

**Table:**

Refinement on *F*^2^	Hydrogen site location: inferred from neighbouring sites
Least-squares matrix: full	H-atom parameters constrained
*R*[*F*^2^ > 2σ(*F*^2^)] = 0.034	*w* = 1/[σ^2^(*F*_o_^2^) + (0.0355*P*)^2^] where *P* = (*F*_o_^2^ + 2*F*_c_^2^)/3
*wR*(*F*^2^) = 0.077	(Δ/σ)_max_ = 0.001
*S* = 1.00	Δρ_max_ = 0.75 e Å^−^^3^
4963 reflections	Δρ_min_ = −0.56 e Å^−^^3^
280 parameters	Extinction correction: none
Primary atom site location: structure-invariant direct methods	Absolute structure: Flack (1983), 2149 Friedel pairs
Secondary atom site location: difference Fourier map	Flack parameter: −0.01 (2)

### Special details

**Table:**

Geometry. All e.s.d.'s (except the e.s.d. in the dihedral angle between two l.s. planes) are estimated using the full covariance matrix. The cell e.s.d.'s are taken into account individually in the estimation of e.s.d.'s in distances, angles and torsion angles; correlations between e.s.d.'s in cell parameters are only used when they are defined by crystal symmetry. An approximate (isotropic) treatment of cell e.s.d.'s is used for estimating e.s.d.'s involving l.s. planes.
Refinement. Refinement of *F*^2^ against ALL reflections. The weighted *R*-factor *wR* and goodness of fit *S* are based on *F*^2^, conventional *R*-factors *R* are based on *F*, with *F* set to zero for negative *F*^2^. The threshold expression of *F*^2^ > σ(*F*^2^) is used only for calculating *R*-factors(gt) *etc*. and is not relevant to the choice of reflections for refinement. *R*-factors based on *F*^2^ are statistically about twice as large as those based on *F*, and *R*- factors based on ALL data will be even larger.

### Fractional atomic coordinates and isotropic or equivalent isotropic displacement parameters (Å^2^)

**Table:**

	*x*	*y*	*z*	*U*_iso_*/*U*_eq_	
Cu1	0.43289 (6)	0.62730 (6)	0.51306 (5)	0.0352 (2)	
Cu2	0.21551 (6)	0.78050 (6)	0.36342 (6)	0.0378 (2)	
I1	0.73774 (3)	0.35628 (3)	0.66369 (3)	0.03691 (12)	
I2	0.56899 (4)	0.12983 (4)	0.53537 (3)	0.04740 (14)	
N1	0.5123 (4)	0.8657 (5)	0.4926 (4)	0.0528 (18)	
H1A	0.5402	0.9200	0.5043	0.063*	
H1B	0.5389	0.8129	0.5106	0.063*	
N2	0.3902 (5)	0.9446 (5)	0.4202 (5)	0.064 (2)	
H2A	0.4189	0.9984	0.4325	0.077*	
H2B	0.3358	0.9442	0.3903	0.077*	
N3	0.3841 (5)	0.3114 (4)	0.4764 (4)	0.0459 (16)	
H3A	0.4073	0.2612	0.5024	0.055*	
H3B	0.3327	0.3064	0.4430	0.055*	
N4	0.5048 (5)	0.3993 (5)	0.5388 (4)	0.0566 (19)	
H4A	0.5263	0.3479	0.5640	0.068*	
H4B	0.5345	0.4534	0.5474	0.068*	
N5	0.2276 (5)	0.5314 (6)	0.6094 (5)	0.067 (2)	
H5A	0.1708	0.5049	0.6202	0.081*	
H5B	0.2535	0.5267	0.5581	0.081*	
N6	0.2313 (5)	0.5839 (5)	0.7483 (4)	0.0615 (19)	
H6A	0.1745	0.5566	0.7571	0.074*	
H6B	0.2602	0.6146	0.7901	0.074*	
N7	−0.0075 (5)	0.5530 (5)	0.2887 (5)	0.057 (2)	
H7A	−0.0649	0.5543	0.2634	0.068*	
H7B	0.0118	0.5018	0.3154	0.068*	
N8	0.0191 (4)	0.7058 (4)	0.2453 (4)	0.0462 (17)	
H8A	−0.0385	0.7056	0.2204	0.055*	
H8B	0.0561	0.7564	0.2431	0.055*	
N9	0.2578 (5)	0.7295 (5)	0.1506 (4)	0.0568 (18)	
H9A	0.2765	0.7019	0.1030	0.068*	
H9B	0.2487	0.6959	0.1972	0.068*	
N10	0.2579 (7)	0.8700 (6)	0.0790 (4)	0.089 (3)	
H10A	0.2767	0.8410	0.0321	0.107*	
H10B	0.2486	0.9314	0.0786	0.107*	
N11	0.1163 (4)	0.9243 (5)	0.6331 (4)	0.0478 (16)	
H11A	0.1405	0.9268	0.6851	0.057*	
H11B	0.0637	0.9570	0.6201	0.057*	
N12	0.2399 (5)	0.8212 (5)	0.5951 (4)	0.065 (2)	
H12A	0.2631	0.8245	0.6474	0.078*	
H12B	0.2691	0.7856	0.5568	0.078*	
N13	0.4435 (6)	0.5766 (7)	0.2168 (5)	0.080 (3)	
S1	0.37489 (12)	0.75471 (12)	0.42179 (11)	0.0289 (4)	
S2	0.38359 (13)	0.49427 (13)	0.43325 (11)	0.0347 (4)	
S3	0.38436 (12)	0.63528 (15)	0.65547 (11)	0.0409 (4)	
S4	0.16498 (11)	0.62484 (13)	0.33684 (12)	0.0377 (4)	
S5	0.20735 (14)	0.88344 (13)	0.24281 (11)	0.0407 (4)	
S6	0.11161 (11)	0.86565 (13)	0.46883 (10)	0.0300 (3)	
C1	0.4293 (5)	0.8635 (5)	0.4470 (4)	0.0371 (16)	
C2	0.4269 (5)	0.3951 (5)	0.4876 (4)	0.0351 (16)	
C3	0.2741 (5)	0.5786 (5)	0.6706 (5)	0.0375 (16)	
C4	0.0505 (4)	0.6292 (5)	0.2864 (4)	0.0339 (15)	
C5	0.2434 (5)	0.8202 (6)	0.1521 (4)	0.0462 (19)	
C6	0.1593 (4)	0.8707 (5)	0.5735 (4)	0.0306 (14)	
C7	0.4660 (6)	0.6535 (9)	0.2169 (5)	0.060 (3)	
C8	0.4976 (7)	0.7529 (7)	0.2179 (7)	0.075 (3)	
H8C	0.4444	0.7925	0.2398	0.112*	
H8D	0.5143	0.7728	0.1595	0.112*	
H8E	0.5551	0.7595	0.2549	0.112*	

### Atomic displacement parameters (Å^2^)

**Table:**

	*U*^11^	*U*^22^	*U*^33^	*U*^12^	*U*^13^	*U*^23^
Cu1	0.0331 (4)	0.0335 (5)	0.0391 (5)	−0.0031 (4)	0.0053 (4)	−0.0024 (4)
Cu2	0.0300 (4)	0.0451 (5)	0.0383 (5)	−0.0036 (4)	−0.0070 (4)	−0.0002 (4)
I1	0.0318 (2)	0.0460 (3)	0.0330 (2)	−0.0026 (2)	0.00101 (19)	−0.0021 (2)
I2	0.0590 (3)	0.0338 (3)	0.0494 (3)	0.0017 (3)	0.0004 (2)	0.0003 (3)
N1	0.041 (3)	0.030 (3)	0.087 (5)	−0.005 (3)	−0.032 (3)	−0.001 (4)
N2	0.049 (4)	0.033 (4)	0.110 (6)	−0.015 (3)	−0.035 (4)	0.013 (4)
N3	0.052 (4)	0.039 (4)	0.047 (4)	−0.001 (3)	−0.006 (3)	0.003 (3)
N4	0.060 (5)	0.043 (4)	0.067 (5)	−0.004 (3)	−0.033 (4)	0.013 (4)
N5	0.042 (4)	0.082 (5)	0.078 (5)	−0.022 (4)	0.012 (4)	−0.004 (4)
N6	0.067 (5)	0.062 (4)	0.055 (4)	−0.004 (4)	0.032 (4)	0.006 (3)
N7	0.047 (4)	0.037 (4)	0.087 (5)	−0.008 (3)	−0.012 (4)	0.008 (4)
N8	0.024 (3)	0.042 (4)	0.072 (5)	0.000 (3)	−0.012 (3)	0.004 (4)
N9	0.058 (4)	0.072 (5)	0.041 (4)	0.015 (4)	0.011 (3)	−0.018 (3)
N10	0.146 (8)	0.089 (6)	0.032 (4)	−0.057 (7)	0.019 (5)	−0.008 (4)
N11	0.042 (3)	0.069 (5)	0.032 (3)	0.018 (3)	−0.006 (3)	−0.016 (3)
N12	0.049 (4)	0.117 (6)	0.028 (3)	0.039 (4)	−0.005 (3)	−0.004 (3)
N13	0.066 (5)	0.106 (7)	0.066 (5)	−0.018 (5)	−0.025 (4)	0.011 (5)
S1	0.0239 (8)	0.0303 (9)	0.0326 (9)	0.0005 (7)	−0.0038 (7)	0.0001 (7)
S2	0.0379 (9)	0.0327 (10)	0.0336 (10)	−0.0020 (8)	−0.0104 (8)	0.0010 (8)
S3	0.0372 (8)	0.0529 (11)	0.0327 (9)	−0.0054 (9)	0.0030 (8)	−0.0016 (9)
S4	0.0291 (8)	0.0365 (10)	0.0474 (10)	0.0026 (7)	−0.0061 (8)	0.0006 (9)
S5	0.0527 (11)	0.0376 (10)	0.0319 (9)	0.0019 (8)	−0.0045 (8)	0.0017 (8)
S6	0.0214 (7)	0.0422 (10)	0.0265 (8)	0.0012 (8)	0.0006 (6)	−0.0039 (9)
C1	0.035 (3)	0.035 (4)	0.042 (4)	0.003 (4)	−0.004 (3)	0.001 (3)
C2	0.032 (4)	0.042 (4)	0.031 (4)	−0.009 (3)	−0.003 (3)	−0.004 (3)
C3	0.038 (4)	0.032 (4)	0.042 (4)	0.007 (3)	0.010 (4)	0.008 (3)
C4	0.030 (3)	0.034 (4)	0.038 (4)	0.001 (3)	0.003 (3)	−0.004 (3)
C5	0.037 (4)	0.068 (6)	0.034 (4)	−0.011 (4)	−0.003 (4)	−0.003 (4)
C6	0.025 (3)	0.042 (4)	0.025 (3)	−0.003 (3)	−0.001 (3)	0.003 (3)
C7	0.040 (5)	0.100 (9)	0.041 (5)	−0.003 (5)	0.000 (4)	0.012 (5)
C8	0.059 (6)	0.096 (8)	0.069 (7)	−0.013 (5)	0.028 (5)	0.000 (6)

### Geometric parameters (Å, °)

**Table:**

Cu1—S3	2.275 (2)	N7—H7B	0.8600
Cu1—S2	2.309 (2)	N8—C4	1.303 (9)
Cu1—S1	2.382 (2)	N8—H8A	0.8600
Cu1—S6^i^	2.411 (2)	N8—H8B	0.8600
Cu2—S4	2.299 (2)	N9—C5	1.273 (10)
Cu2—S5	2.335 (2)	N9—H9A	0.8600
Cu2—S1	2.341 (2)	N9—H9B	0.8600
Cu2—S6	2.435 (2)	N10—C5	1.328 (10)
N1—C1	1.313 (8)	N10—H10A	0.8600
N1—H1A	0.8600	N10—H10B	0.8600
N1—H1B	0.8600	N11—C6	1.309 (8)
N2—C1	1.307 (9)	N11—H11A	0.8600
N2—H2A	0.8600	N11—H11B	0.8600
N2—H2B	0.8600	N12—C6	1.321 (9)
N3—C2	1.307 (8)	N12—H12A	0.8600
N3—H3A	0.8600	N12—H12B	0.8600
N3—H3B	0.8600	N13—C7	1.109 (12)
N4—C2	1.306 (8)	S1—C1	1.719 (7)
N4—H4A	0.8600	S2—C2	1.708 (7)
N4—H4B	0.8600	S3—C3	1.689 (7)
N5—C3	1.301 (10)	S4—C4	1.718 (6)
N5—H5A	0.8600	S5—C5	1.711 (8)
N5—H5B	0.8600	S6—C6	1.725 (6)
N6—C3	1.321 (9)	S6—Cu1^ii^	2.411 (2)
N6—H6A	0.8600	C7—C8	1.442 (14)
N6—H6B	0.8600	C8—H8C	0.9600
N7—C4	1.313 (8)	C8—H8D	0.9600
N7—H7A	0.8600	C8—H8E	0.9600
			
S3—Cu1—S2	117.58 (8)	C6—N11—H11A	120.0
S3—Cu1—S1	115.55 (8)	C6—N11—H11B	120.0
S2—Cu1—S1	100.97 (8)	H11A—N11—H11B	120.0
S3—Cu1—S6^i^	99.89 (6)	C6—N12—H12A	120.0
S2—Cu1—S6^i^	112.14 (7)	C6—N12—H12B	120.0
S1—Cu1—S6^i^	111.15 (7)	H12A—N12—H12B	120.0
S4—Cu2—S5	114.90 (8)	C1—S1—Cu2	109.7 (2)
S4—Cu2—S1	101.08 (7)	C1—S1—Cu1	112.4 (2)
S5—Cu2—S1	115.93 (7)	Cu2—S1—Cu1	129.34 (8)
S4—Cu2—S6	113.86 (7)	C2—S2—Cu1	106.8 (2)
S5—Cu2—S6	101.49 (8)	C3—S3—Cu1	111.0 (3)
S1—Cu2—S6	110.05 (7)	C4—S4—Cu2	108.0 (3)
C1—N1—H1A	120.0	C5—S5—Cu2	108.3 (3)
C1—N1—H1B	120.0	C6—S6—Cu1^ii^	105.0 (2)
H1A—N1—H1B	120.0	C6—S6—Cu2	115.0 (2)
C1—N2—H2A	120.0	Cu1^ii^—S6—Cu2	131.52 (7)
C1—N2—H2B	120.0	N2—C1—N1	119.0 (7)
H2A—N2—H2B	120.0	N2—C1—S1	121.1 (5)
C2—N3—H3A	120.0	N1—C1—S1	119.9 (6)
C2—N3—H3B	120.0	N4—C2—N3	117.9 (6)
H3A—N3—H3B	120.0	N4—C2—S2	121.8 (5)
C2—N4—H4A	120.0	N3—C2—S2	120.2 (5)
C2—N4—H4B	120.0	N5—C3—N6	117.9 (7)
H4A—N4—H4B	120.0	N5—C3—S3	123.7 (6)
C3—N5—H5A	120.0	N6—C3—S3	118.5 (6)
C3—N5—H5B	120.0	N8—C4—N7	118.6 (6)
H5A—N5—H5B	120.0	N8—C4—S4	122.2 (5)
C3—N6—H6A	120.0	N7—C4—S4	119.2 (6)
C3—N6—H6B	120.0	N9—C5—N10	118.6 (8)
H6A—N6—H6B	120.0	N9—C5—S5	124.3 (6)
C4—N7—H7A	120.0	N10—C5—S5	117.1 (7)
C4—N7—H7B	120.0	N11—C6—N12	118.8 (6)
H7A—N7—H7B	120.0	N11—C6—S6	120.3 (5)
C4—N8—H8A	120.0	N12—C6—S6	120.8 (5)
C4—N8—H8B	120.0	N13—C7—C8	178.6 (11)
H8A—N8—H8B	120.0	C7—C8—H8C	109.5
C5—N9—H9A	120.0	C7—C8—H8D	109.5
C5—N9—H9B	120.0	H8C—C8—H8D	109.5
H9A—N9—H9B	120.0	C7—C8—H8E	109.5
C5—N10—H10A	120.0	H8C—C8—H8E	109.5
C5—N10—H10B	120.0	H8D—C8—H8E	109.5
H10A—N10—H10B	120.0		

Symmetry codes: (i) *x*+1/2, −*y*+3/2, −*z*+1; (ii) *x*−1/2, −*y*+3/2, −*z*+1.

### Hydrogen-bond geometry (Å, °)

**Table:**

*D*—H···*A*	*D*—H	H···*A*	*D*···*A*	*D*—H···*A*
N1—H1A···I2^iii^	0.86	2.97	3.799 (7)	161
N1—H1B···S6^i^	0.86	2.68	3.524 (7)	167
N2—H2A···I2^iii^	0.86	3.14	3.929 (6)	154
N2—H2B···S5	0.86	2.96	3.752 (7)	154
N2—H2B···I1^ii^	0.86	3.17	3.667 (7)	119
N3—H3A···I2	0.86	2.87	3.645 (6)	150
N3—H3B···I1^iv^	0.86	3.06	3.720 (6)	135
N4—H4A···I2	0.86	3.11	3.837 (7)	144
N4—H4A···I1	0.86	3.22	3.706 (6)	119
N4—H4B···S6^i^	0.86	2.73	3.563 (7)	165
N5—H5A···N13^v^	0.86	2.41	3.192 (10)	152
N5—H5A···I2^iv^	0.86	3.32	3.796 (8)	118
N7—H7A···I1^vi^	0.86	3.04	3.839 (7)	156
N7—H7B···I2^iv^	0.86	3.02	3.837 (7)	159
N8—H8A···I1^vi^	0.86	2.93	3.759 (6)	161
N8—H8B···S5	0.86	2.69	3.526 (6)	166
N9—H9A···I2^vii^	0.86	3.12	3.922 (6)	155
N9—H9B···S4	0.86	2.61	3.429 (7)	161
N10—H10A···I1^vii^	0.86	3.01	3.716 (7)	141
N11—H11A···I1^viii^	0.86	2.99	3.791 (6)	155
N11—H11B···S2^ii^	0.86	2.63	3.466 (6)	163
N12—H12A···I1^viii^	0.86	2.92	3.732 (6)	158
N12—H12B···S1	0.86	2.54	3.338 (6)	155
N6—H6A···S2^v^	0.86	2.89	3.397 (6)	119
N6—H6A···N13^v^	0.86	2.51	3.266 (11)	147

Symmetry codes: (iii) *x*, *y*+1, *z*; (i) *x*+1/2, −*y*+3/2, −*z*+1; (ii) *x*−1/2, −*y*+3/2, −*z*+1; (iv) *x*−1/2, −*y*+1/2, −*z*+1; (v) −*x*+1/2, −*y*+1, *z*+1/2; (vi) −*x*+1/2, −*y*+1, *z*−1/2; (vii) −*x*+1, *y*+1/2, −*z*+1/2; (viii) −*x*+1, *y*+1/2, −*z*+3/2.
